# Preparation and Characterization of Novel Polyvinylidene Fluoride/2-Aminobenzothiazole Modified Ultrafiltration Membrane for the Removal of Cr(VI) in Wastewater

**DOI:** 10.3390/polym10010019

**Published:** 2017-12-25

**Authors:** Xiuju Wang, Kaili Zhou, Zhun Ma, Xingjie Lu, Liguo Wang, Zhongpeng Wang, Xueli Gao

**Affiliations:** 1 Key Laboratory of Water Resources and Environmental Engineering in Universities of Shandong, University of Jinan, Jinan 250022, China; 15318835373@163.com (K.Z.); 18764126632@163.com (X.L.); 2 School of Resources and Environment, University of Jinan, Jinan 250022, China; 3 Shandong Provincial Engineering Technology Research Center for Ecological Carbon Sink and Capture Utilization, Jinan 250022, China; 4 College of Chemical and Environmental Engineering, Shandong University of Science and Technology, Qingdao 266590, China; mzyxy199@163.com; 5 Key Laboratory of Marine Chemistry Theory and Technology, Ministry of Education, College of Chemistry and Chemical Engineering, Ocean University of China, Qingdao 266100, China; gaoxueli816@ouc.edu.cn

**Keywords:** polyvinylidene fluoride, 2-aminobenzothiazole, ultrafiltration membrane, chromium ion

## Abstract

Hexavalent chromium is one of the main heavy metal pollutants. As the environmental legislation becomes increasingly strict, seeking new technology to treat wastewater containing hexavalent chromium is becoming more and more important. In this research, a novel modified ultrafiltration membrane that could be applied to adsorb and purify water containing hexavalent chromium, was prepared by polyvinylidene fluoride (PVDF) blending with 2-aminobenzothiazole via phase inversion. The membrane performance was characterized by evaluation of the instrument of membrane performance, infrared spectroscopy (FTIR), scanning electron microscope (SEM), and water contact angle measurements. The results showed that the pure water flux of the PVDF/2-aminobenzothiazole modified ultrafiltration membrane was 231.27 L/m^2^·h, the contact angle was 76.1°, and the adsorption capacity of chromium ion was 157.75 µg/cm^2^. The PVDF/2-aminobenzothiazole modified ultrafiltration membrane presented better adsorption abilities for chromium ion than that of the traditional PVDF membrane.

## 1. Introduction

Today, the emission of heavy metals into the aquatic and marine water systems has attracted increasing concerns, because these emissions create environmental issues that are associated with human health. With rapid development of society and economy, large quantities of wastewater containing heavy metals are generated from industries, including textiles, leather tanning, electroplating, steel making, metal finishing, pigment manufacture, wood preservatives, as well as in mine tailings [1,2]. Chromium ion is one of the major toxic heavy metals, especially hexavalent chromium. The methods of dealing with wastewater containing chromium include chemical reduction precipitation [3], electrolytic process [4], biological method [5], adsorption method [6], and ion exchange method [7]. However, all of the above methods have disadvantages to a certain degree, such as high costs, use of toxic compounds, large space requirements for installation, generation of secondary pollutants, and others.

Nowadays, a number of technologies have been introduced to deal with Cr(VI) in water, such as adsorption, chemical precipitation, ion-exchange, nanofiltration, and ecological remediation [8,9,10,11]. Membrane separation techniques act as an emerging technology in the 21st century [12]. It is witnessing an era of rapid growth, due to continuous research and development in both the academic and private industry [13]. Additionally, the membrane technology has also been introduced as an efficient technique for the treatment of chromium-containing wastewater. Membrane separation technology includes microfiltration (MF), ultrafiltration (UF), nanofiltration (NF), reverse osmosis (RO), and liquid membrane permeation. Most of these methods have been used extensively for the treatment of heavy metal wastewater [14]. For instance, wastewater that contains Cu^2+^, Cd^2+^, Ni^2+^, Cr^2+^ was treated by nanofiltration and reverse osmosis technology [15,16].

Ultrafiltration, a membrane technology for wastewater treatment, has received increasing attention in recent years. It has been extensively used as a separation technique in the dairy industry [17]. Ultrafiltration membrane technology in the treatment of heavy metal ions has been shown to be an effective and cheap approach, not only could it improve general water quality [18,19,20,21], but also, it could control membrane fouling [22,23,24,25,26]. On the other hand, membrane separation techniques have shown great promise for metal ion removal, due to their high efficiency, space saving, low cost, and easy operation. This could help enhance membrane management in real practice [27,28,29,30].

The objective of this study is to investigate how and why the synthesis of 2-aminobenzothiazole modified membranes significantly enhances aspects of membrane ultrafiltration ability and adsorption capacity. Polyvinylidene fluoride (PVDF) membranes remain popular in many applications, due to their excellent chemical resistance, and good thermal and mechanical properties. Given that 2-aminobenzothiazole has adsorption capacity for chromium ions, 2-aminobenzothiazole was used to modify the polyvinylidene fluoride (PVDF) membrane in this paper, and a type of modified PVDF membrane for hexavalent chromium adsorption and purification was prepared. The mechanism of this method was that 2-aminobenzothiazole contains some functional groups, such as =C–H and H–C–H, which could generate clathrate with chromium ions. Those functional groups could improve the affinity between modified ultrafiltration membrane and chromium ions. The method in this paper of blending 2-aminobenzothiazole during membrane preparation to improve the adsorbability, has never been reported. The tests were performed under several operating conditions: the main parameters investigated were the pure water flux, and the adsorption capacity of chromium ion.

## 2. Experimental Procedure

### 2.1. Materials

PVDF was purchased from Shanghai San Aifu New Chemical Materials Co. (Shanghai, China), *N*,*N*-dimethyl acetamide(DMAc) was obtained from Beijing Yili fine chemicals Co. (Beijing, China), 2-aminobenzothiazole and 1,5-diphenylcarbazide (DPCI) were obtained from Shanghai JingChun biological technology Co., Ltd. (Shanghai, China), Tween 80 was purchased from Sinopharm Chemical Reagent Co.,Ltd. (Shanghai, China), Polyethylene glycol (PEG-400) was purchased from Tianjin Damao chemical reagent factory (Tianjin, China). All the chemicals were of analytical grade.

### 2.2. Preparation of PVDF/2-Aminobenzothiazole Modified Ultrafiltration Membrane

PVDF was dried at 70 °C in the oven for 24 h before further utilization. A certain amount of solvent (DMAc) and 2-aminobenzothiazole were put into a flask, then PEG-400 (11.0 wt %) and Tween-80 (1.0 wt %) were added in. The reaction was conducted at 70 °C in the water bath for 6 h, and the flask containing casting solution was defoamed, in situ, for 12 h at room temperature. Then, the casting membrane solution was conducted into a clean glass plate to prepare ultrafiltration membrane, and was put into deionized water for 24 h. The deionized water was changed every 3 h, and then the membrane was dried for later experiments.

### 2.3. Characterization of PVDF/2-Aminobenzothiazole Modified Ultrafiltration Membrane

For all the experiments, temperature and barometric pressure were 25 °C and 0.1 MPa, respectively.

The pure water flux of the blend membrane can be calculated according to the following equation:

(1) $$J_{w}=\frac{V}{A\cdot t}$$

where *J*_w_ was pure water flux of the blend membrane (L/m^2^·h), *V* was the pure water through the membrane volume within half an hour (L), *t* was the time (usually refers to half an hour, h), and *A* was the area of the modified membrane (cm^2^).

Bovine serum albumin (BSA) was used frequently as a standard substance to evaluate the protein rejection performance of the prepared membrane. In this experiment, a stable laboratory state was maintained—the same as the conditions for determination of pure water flux. The initial concentration of BSA was 1.0 g/L. The absorbance was determined at the distance of 280 nm with a UV visible spectrophotometer, then calculated by the following formula:

(2) $$R=\left(1-\frac{C_{b}}{C_{a}}\right)\times 100\%$$

Here, *R* was the retention rate (%), *C*_b_ was the concentration of BSA in the filtrate (g/L), and *C*_a_ was the concentration of BSA at initial times (g/L).

The water contact angles of the membranes were measured using a contact angle goniometer (OCA40, Dataphysics, Stuttgart, Germany) at room temperature. To minimize the experimental errors, the contact angle was measured at least three times for each sample, and then the average was reported.

To confirm the chemical modification, the FTIR spectra of the ultrafiltration membranes were recorded by using a Bruker instrument (VERTEX-70, Bruker, Billerica, MA, USA). Each spectrum was captured by averaging 64 scans at a resolution of 4 cm^−1^. The structural morphology of the surface and a cross section of the membrane were studied by SEM (JEOL Model S-4800, Tokyo, Japan). Samples of the membranes were frozen in liquid nitrogen and then fractured. All results were measured at least three times.

The amount of chromium ion adsorbed on membranes was one of the most important indexes in evaluating the removal ability of heavy metals of the membranes. In this process, the initial concentration of chromium ion solution was 100 mg/L. The pH was adjusted to 7.0, and the temperature was controlled at 25 °C. “Photometry of Diphenylcarbazide” was used to measure the concentration of chromium ion. Adsorption experiments for chromium ion were carried out by varying the adsorption time (2, 4, 6, and 8 h). The relationship between absorbance and chromium ion concentration obtained from the chromium ion concentration standard curve, and the relational expression, is *A* = 0.3023*C*x − 0.0189, *R*^2^ = 0.9992. The formula of adsorbed chromium is described as follows:

(3) $$P=\frac{\left(C_{0}-\frac{A+0.0189}{0.3023}\right)}{m}\times 0.1$$

where *P* was adsorbance of the modified membrane (mg/cm^2^), *C*_0_ was the concentration of chromium ion at initial times (mg/L), *A* was the absorbance value of the chromium ion, and *m* was the area of the modified membrane (cm^2^).

Desorption process was carried out in hydrochloric acid solution (0.5 mol/L), and the chromium ion concentration of the stripping liquid was measured at certain periods of time, until the concentration of chromium ion was approximately steady. The adsorption performance of blend membranes was investigated after rinsing with deionized water. The rinsing process was repeated 3 times.

## 3. Results and Discussion

### 3.1. Effect of 2-Aminobenzothiazole Dosage on Membrane Performance

**Figure 1:**
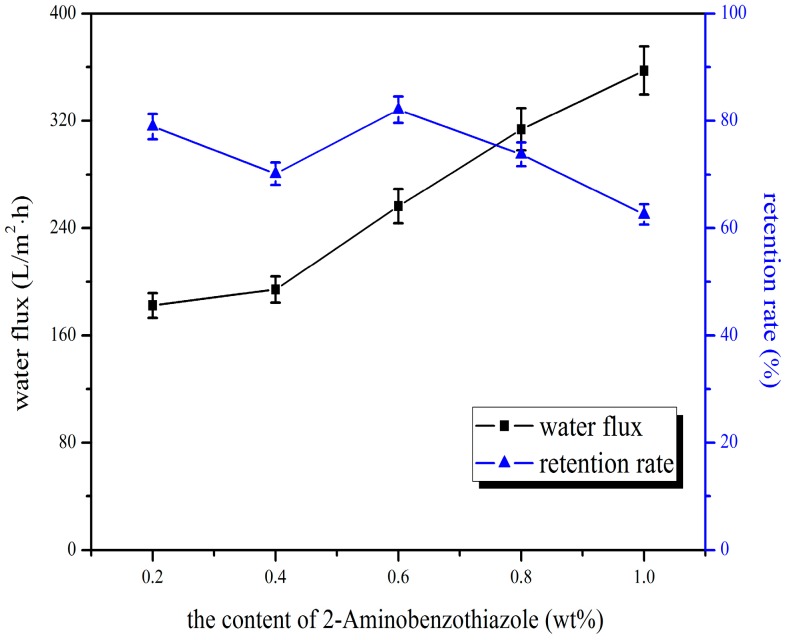
Effect of 2-aminobenzothiazole dosage on water flux and retention rate of bovine serum albumin (BSA).

**Figure 2:**
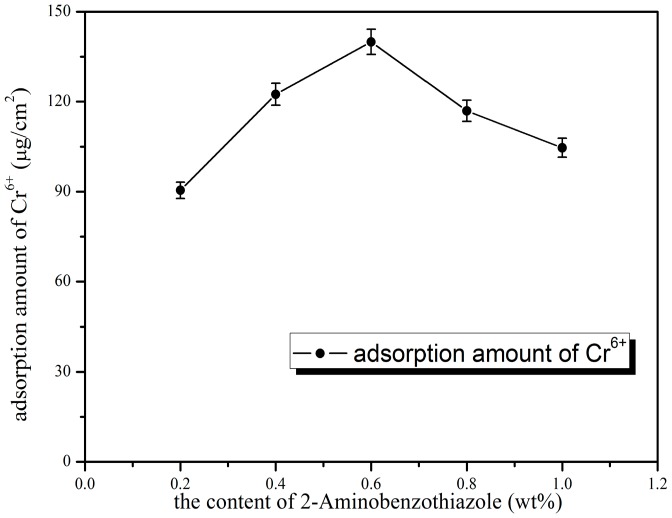
Effect of 2-aminobenzothiazole dosage on adsorption amount of Cr^6+^.

As could be seen from the Figure 1, the pure water flux increased considerably with the dosage of 2-aminobenzothiazole into the blend membrane. This was because higher dosage of 2-aminobenzothiazole resulted in the increase of the porosity of the membrane. However, the higher 2-aminobenzothiazole dosage did not have significant effects on increasing the retention rate. This was due to 2-aminophenylmethythiazole not being a pore-forming agent, and could therefore not increase the pore diameter of the separation membrane. Figure 2 represents the effect of 2-aminobenzothiazole dosage on adsorption amount of Cr^6+^, and the results illustrate that chromium ion adsorption increased at first, and then decreased with the rising of 2-aminobenzothiazole. This was due to the agglomeration and decreased effective surface of the active groups in the high blending ratio, which led to reduction of the membrane surface functional groups. Considered overall, 0.6 wt % of 2-aminobenzothiazole was chosen as the best dosage, the adsorption quantity was 139.95 μg/cm^2^, the pure water flux was 256.37 L/m^2^·h, and the retention rate was 82.09%.

### 3.2. Effect of Blending Time on Membrane Performance

**Figure 3:**
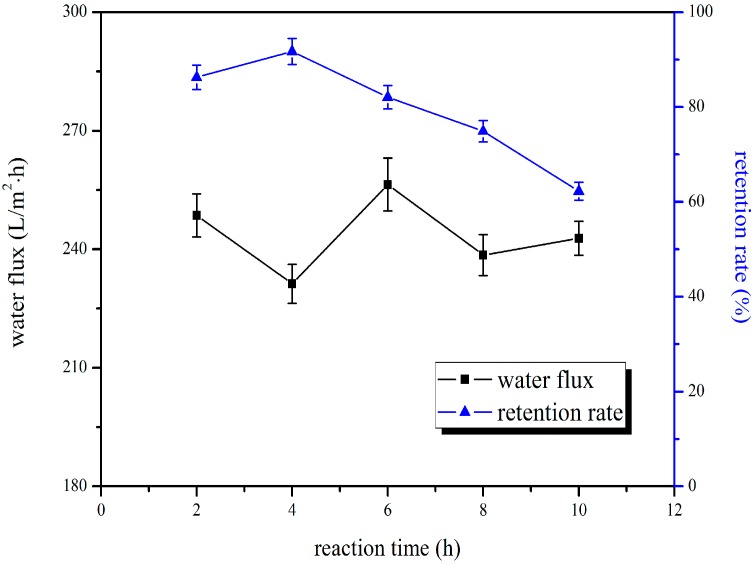
Effect of blending time on water flux and retention rate of BSA.

**Figure 4:**
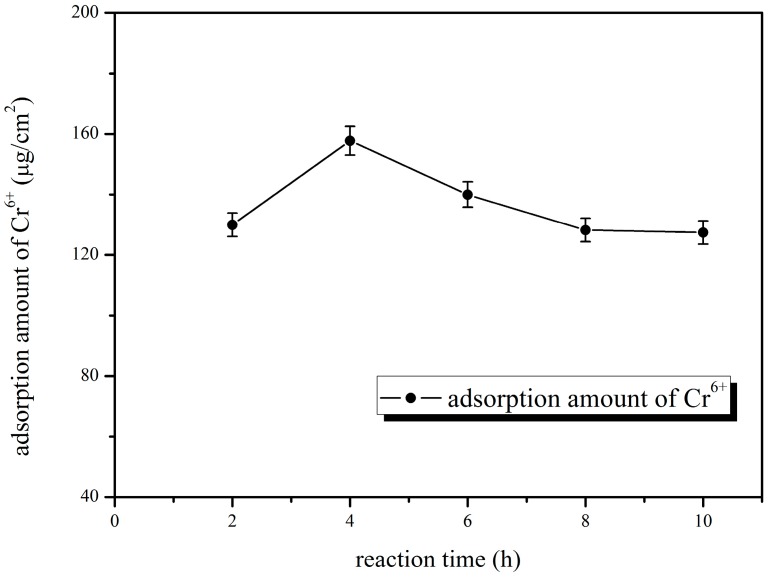
Effect of blending time on adsorption amount of Cr^6+^.

As shown in Figure 3, there was no significant change in the pure water flux of blend membrane with prolonged blending time, and its retention rate showed a tendency to decrease after increasing at first. Figure 4 showed that chromium ion adsorption increased prominently in the first few minutes of operation, and then decreased at later times. This was due to the 2-aminobenzothiazole becoming more uniformly distributed in the membrane casting solution as blending time progressed, which was beneficial to the adsorption of chromium ion. However, after a certain time, the continuous heating did not produce a good effect on the uniformity of the casting membrane solution, but adversely affected uniformity of the casting membrane solution, and resulted in the attenuation of membrane performance. This is consistent with the consensus of casting liquid preparation technology and the energy requirement in membrane industry. So, 4 h was chosen as the optimal blending time, and the adsorption quantity was 157.75 μg/cm^2^, and the pure water flux and retention rate were 231.27 L/m^2^·h and 91.71%, respectively.

### 3.3. Effect of Blending Temperature on Membrane Performance

**Figure 5:**
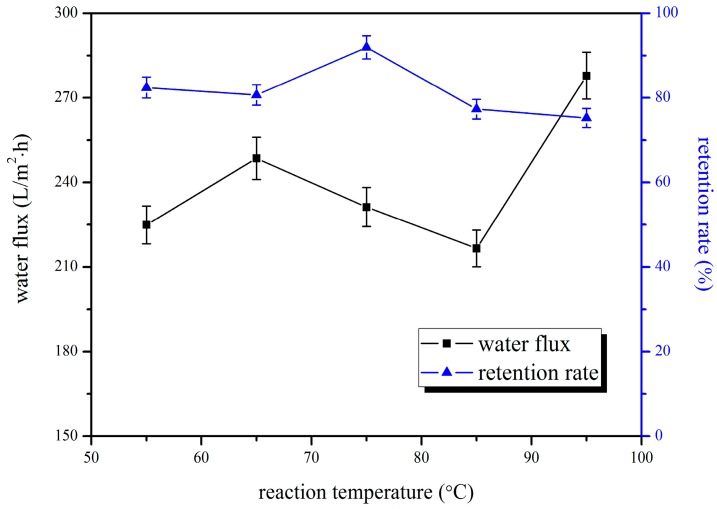
Effect of blending temperature on water flux and retention rate of BSA.

**Figure 6:**
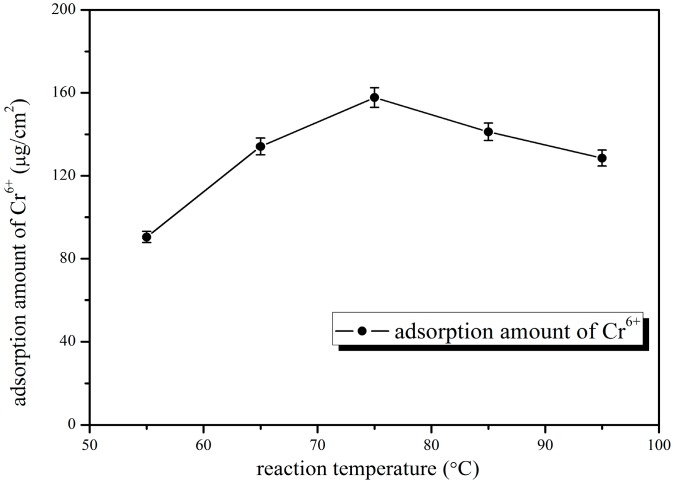
Effect of blending temperature on adsorption amount of Cr^6+^.

As can be seen from Figure 5, with the increase of blending temperature, the retention rate of the blend ultrafiltration membrane showed a tendency to decrease after increasing at first, but the pure water flux presented a trend of decreasing after increasing, and then increasing, then the pure water flux was the largest when the blending temperature was 95 °C, while the retention rate of bovine serum albumin was minimum. This was because that the high temperature at this time caused the pore diameter of the membrane to become larger, and resulted in a sharp increase in pure water flux, and a significant decrease in the retention rate. When the blending temperature was 75 °C, the retention rate of membrane reached a maximum, the pure water flux was better, so we chose 75 °C as the optimal blending temperature. As can be seen from Figure 6, the chromium ion adsorption showed a similar consequence: it increased at first, and then decreased with the increasing of blending temperature. This was because of the structural change of the blend membrane and the reduction of functional groups resulting from high temperature, which decreased their adsorption capacities. Taking into account all these factors, we confirmed that 75 °C was the optimal blending temperature. The adsorption quantity was 157.75 μg/cm^2^, the pure water flux was 231.27 L/m^2^·h, and the retention rate was 91.97% at that moment.

### 3.4. Determination of the Optimal Preparation Technique of the Novel Modified Ultrafiltration Membrane

According to the influence of various factors on the PVDF/2-aminobenzothiazole modified ultrafiltration membrane, the optimal conditions for the preparation of modified ultrafiltration membrane were determined: 2-aminobenzothiazole content was 0.6 wt %, and the blending time and temperature were 4 h and 75 °C, respectively. In this case, the pure water flux of the modified ultrafiltration membrane was 231.27 L/m^2^·h, and its retention rate of BSA was 91.71%, and its adsorption capacity of chromium ion was 157.75 µg/cm^2^.

### 3.5. Water Contact Angle Analysis

Better hydrophilicity promotes the membrane to form hydrogen bonds with water molecules, which allows for formation of a protection layer on the membrane surface. The membrane fouling will be alleviated because of the reduction of adsorption of protein and other organic compounds in the membrane surface. The contact angle of the traditional PVDF membrane was 79.3°. The contact angle of the PVDF/2-aminobenzothiazole modified ultrafiltration membrane was 76.1°, which was lower than that of the traditional PVDF membrane. Contact angle could reflect the hydrophilicity of the membrane; as the contact angle increases, the hydrophilic property reduces. Therefore, the modified ultrafiltration membrane had better hydrophilic properties than the unmodified membrane. The results indicated that 2-aminophenylprothiazole could not only adsorb hexavalent chromium ions, but also improve the hydrophilicity of PVDF ultrafiltration membrane.

### 3.6. Fourier Transform Infrared Spectroscopy (FTIR)

**Figure 7:**
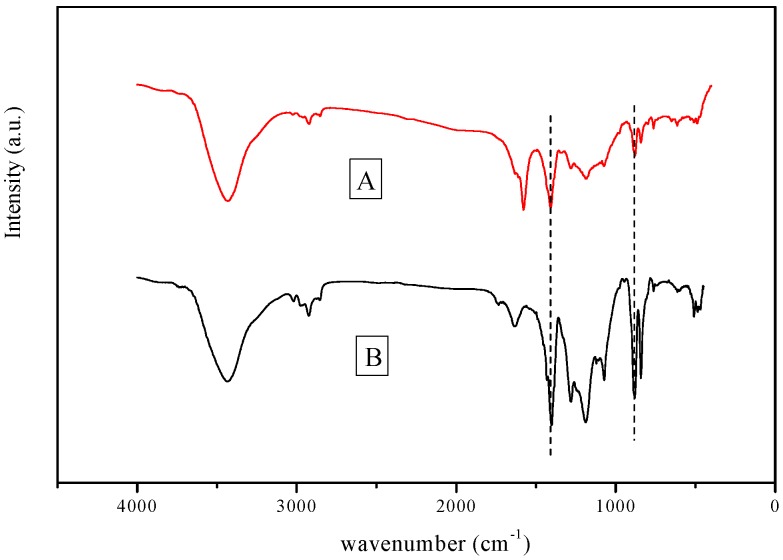
The FTIR image of the membrane. **A** stands for unmodified PVDF ultrafiltration membrane; **B** stands for PVDF modified ultrafiltration membrane.

As shown in Figure 7, the adsorptions between 3000 and 3100 cm^−1^, which stand for a characteristic vibration adsorption frequency of unsaturated hydrocarbon key (=C–H), were stronger than that of the unmodified membranes. Modified membranes’ characteristic peaking in the range 800–1000 cm^−1^ shows the presence of C–N key. Compared with the unmodified membrane, the PVDF/2-aminobenzothiazole modified membrane showed stronger adsorption at 1450 cm^−1^, which could be especially attributed to the H–C–H of 2-aminobenzothiazole. These data indicated that the 2-aminobenzothiazole has been successfully blended into the PVDF modified membrane.

### 3.7. Scanning Electron Microscope (SEM) Analysis

**Figure 8:**
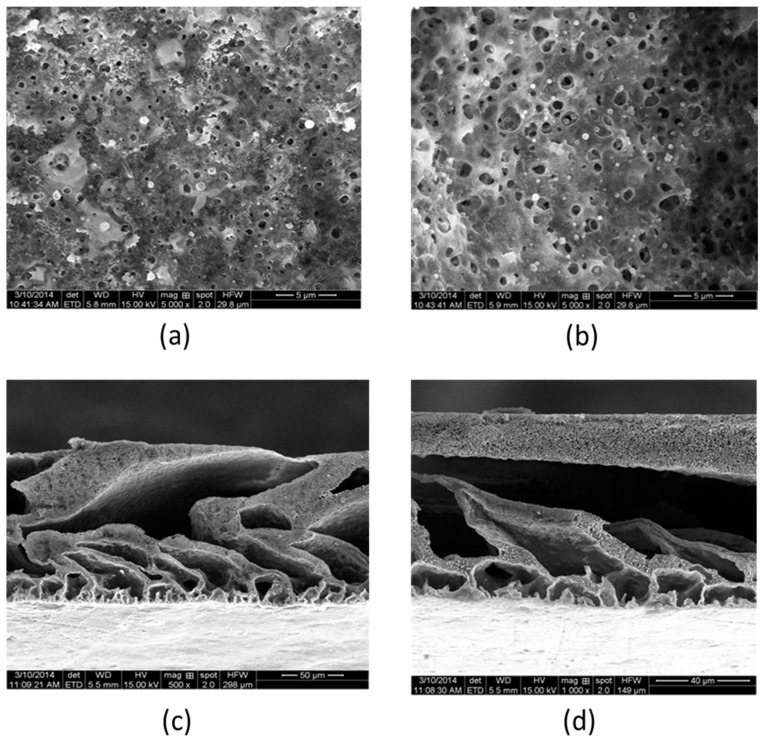
The SEM image of membrane. (**a**) Surface of PVDF/2-aminobenzothiazole modified membrane; (**b**) surface of traditional PVDF membranes; (**c**) cross section of PVDF/2-aminobenzothiazole modified membrane; and (**d**) cross section of traditional PVDF membrane.

In order to assess the structure characteristics of PVDF/2-aminobenzothiazole modified membranes and traditional membranes, the SEM images of the surface and the cross section were shown in Figure 8a–d. The SEM images provided qualitative information which showed that the bore diameter of the surface image and the number were smaller than traditional membranes, and the cross-section image of PVDF/2-aminobenzothiazole modified membrane showed that the corticals were denser. On the other side, the pores of modified membranes were smaller than traditional membranes. In short, the water flux of PVDF/2-aminobenzothiazole modified membrane was lower than traditional membranes, but the BSA retention rate was improved.

### 3.8. Performance Analysis of PVDF Modified Membrane Adsorption for Chromium Ion

As can be seen from Table 1, the initial adsorption capacity for chromium ion of the PVDF/2-aminobenzothiazole membrane was 157 µg/cm^2^, and the pure water flux also dropped from 231 to 184 L/m^2^·h. The downward trend in pure water flux may be due to the membrane being polluted during adsorption and desorption. In the latter three adsorption values, we concluded that the adsorption capacities were 94.91, 89.81, and 85.99%, respectively, by using the counting steps system. Adsorption capacity did not reach 100% because a part of the chromium ion possibly resided in the membrane pore.

## 4. Conclusions

In this paper, the influence of various factors on the PVDF/2-aminobenzothiazole modified ultrafiltration membrane were investigated, and the optimal conditions for the preparation were determined. Based on the experiments conducted here, the following conclusions could be drawn:

(1) The results obtained indicated that chromium could be successfully removed from the waste water by modified ultrafiltration membrane, due to its high adsorption capacities, and the optimal ultrafiltration membrane was obtained when 2-aminobenzothiazole content was 0.6 wt %, the blending time, and temperature was 4 h and 75 °C, respectively. In this case, its pure water flux was 231.27 L/m^2^·h, and the BSA rejection rate was 91.71%, its adsorption capacity of chromium ion was 157.75 µg/cm^2^.

(2) By studying the change of the contact angle, we could confirm that the contact angle of PVDF/2-aminobenzothiazole modified ultrafiltration membrane was smaller, but the hydrophilicity of modified ultrafiltration membrane was higher than the traditional membrane.

Although this paper drew the conclusion that 2-aminobenzothiazole blended with PVDF was feasible for the using in removal of hexavalent chromium ion, the pure water flux was not high enough, and even lower than the traditional ultrafiltration membrane. Therefore, we need to find a way to enhance the pure water flux of the modified ultrafiltration membrane in further research.

## Figures and Tables

**Table 1:** The adsorption/desorption of polyvinylidene fluoride (PVDF) modified membrane and pure water flux.

Adsorption Times	Pure Water Flux (L/m^2^·h)	Amount of Adsorption (µg/cm^2^)
1	231	157
2	209	149
3	197	141
4	184	135
